# *Pseudomonas aeruginosa N*-3-oxo-dodecanoyl-homoserine Lactone Elicits Changes in Cell Volume, Morphology, and AQP9 Characteristics in Macrophages

**DOI:** 10.3389/fcimb.2016.00032

**Published:** 2016-03-24

**Authors:** Angelika Holm, Karl-Eric Magnusson, Elena Vikström

**Affiliations:** Department of Clinical and Experimental Medicine, Faculty of Medicine and Health Sciences, Linköping UniversityLinköping, Sweden

**Keywords:** host-bacteria interactions, quorum sensing, *N*-acylhomoserine lactone, innate immunity, macrophage, water homeostasis, aquaporin

## Abstract

Quorum sensing (QS) communication allows *Pseudomonas aeruginosa* to collectively control its population density and the production of biofilms and virulence factors. QS signal molecules, like *N*-3-oxo-dodecanoyl-*L*-homoserine lactone (3O-C_12_-HSL), can also affect the behavior of host cells, e.g., by modulating the chemotaxis, migration, and phagocytosis of human leukocytes. Moreover, host water homeostasis and water channels aquaporins (AQP) are critical for cell morphology and functions as AQP interact indirectly with the cell cytoskeleton and signaling cascades. Here, we investigated how *P. aeruginosa* 3O-C_12_-HSL affects cell morphology, area, volume and AQP9 expression and distribution in human primary macrophages, using quantitative PCR, immunoblotting, two- and three-dimensional live imaging, confocal and nanoscale imaging. Thus, 3O-C_12_-HSL enhanced cell volume and area and induced cell shape and protrusion fluctuations in macrophages, processes tentatively driven by fluxes of water across cell membrane through AQP9, the predominant AQP in macrophages. Moreover, 3O-C_12_-HSL upregulated the expression of AQP9 at both the protein and mRNA levels. This was accompanied with enhanced whole cell AQP9 fluorescent intensity and redistribution of AQP9 to the leading and trailing regions, in parallel with increased cell area in the macrophages. Finally, nanoscopy imaging provided details on AQP9 dynamics and architecture within the lamellipodial area of 3O-C_12_-HSL-stimulated cells. We suggest that these novel events in the interaction between *P. aeruginosa* and macrophage may have an impact on the effectiveness of innate immune cells to fight bacteria, and thereby resolve the early stages of infections and inflammations.

## Introduction

The ability of a human cell to adjust and adapt its volume is vital for survival and proper functions. The aquaporins (AQP) are membrane-spanning water channels that facilitate the transport of water and small uncharged solutes over the plasma membrane (Verkman, 2005; Benga, 2012). By interacting with the cytoskeleton and signaling cascades, the AQP can influence cell morphology, volume, movement and migration (Saadoun et al., 2005; Karlsson et al., 2011, 2013). Today, 13 human AQP, AQP0-AQP12 are known and divided into three subfamilies based on their pore-selectivity. The first one is comprised of the water-specific channels being permeable only to water; the second subfamily, aquaglyceroporins allows transport of both water and small uncharged molecules, like glycerol; the super-AQP, or subcellular-AQP (S-AQP) make up the third subgroup (Benga, 2012; Verkman, 2005).

In human leukocytes, the aquaglyceroporin AQP9 is the most abundant among the AQP. It localizes at the leading edge in moving cells (Loitto et al., 2007; Karlsson et al., 2011), and is critical for migration of neutrophils and epithelial cells (Karlsson et al., 2011) and metabolism (Karlsson et al., 2011; Ana et al., 2014; Leire et al., 2014). There is evidence that AQP9 is upregulated in blood leukocytes after *in vivo* endotoxin stimulation in humans (Talwar et al., 2006). Moreover, in patients with infectious endocarditis (Thuny et al., 2012), and systemic inflammatory response syndrome (Matsushima et al., 2014), the expression of AQP9 was elevated compared with healthy controls. Taken together, these data suggest an involvement of AQP9 in the inflammatory processes during bacterial infections.

The Gram-negative bacterium *Pseudomonas aeruginosa* is an opportunistic pathogen that forms biofilms and causes acute and chronic infections, typically in critically ill patients. Using a small molecule-based communication system, quorum sensing (QS), bacteria can sense their population density and collectively regulate the expression of multiple genes encoding the production of virulence factors and biofilms that enhance the pathogenicity and survival of bacteria (de Kievit, 2009; Rutherford and Bassler, 2012). The *N*-acylhomoserine lactones (AHL)-dependent QS-system allows *P. aeruginosa* to produce and recognize two diffusible AHL, *N*-butyryl-*L*-homoserine lactone (C_4_-HSL) with short fatty-acid chain and *N*-3-oxo-dodecanoyl-*L*-homoserine lactone (3O-C_12_-HSL) with long fatty-acid chain. In these processes, the AHL-dependent QS-system controls transcription and production of extracellular virulence factors, like lectins, elastases, proteases, exotoxin A and pyocyanin, and also surface-active rhamnolipids important in the late stage of biofilm development (Venturi, 2006). Together, these factors help bacteria collectively adhere, swarm, colonize, and destroy host cells and tissues which may result in a more severe outcome of infections and inflammations and development of disease.

Through inter-kingdom signaling, the AHL can also interact with host cells, including epithelial cells that normally form physical barriers to pathogens, and cells of the innate and adaptive immune system (Pacheco and Sperandio, 2009). The AHL molecules are strong chemoattractants for leukocytes and increase migration (Karlsson et al., 2012) and phagocytosis in both neutrophils (Wagner et al., 2007), and macrophages (Vikström et al., 2005). Long fatty-acid chain AHL may disrupt epithelial barriers and modulate epithelial cell migration in a dose- and time-dependent manner (Vikström et al., 2006, 2010; Karlsson et al., 2012). Moreover, the expression and secretion of different pro- and anti-inflammatory cytokines in host cells are influenced by the bacterial AHL (Telford et al., 1998; Smith et al., 2001, 2002; Hooi et al., 2004). During a *P. aeruginosa* infection the innate immune cells can migrate toward the site of infection but in close proximity to bacterial biofilms their functions are inhibited rather than stimulated by enhanced concentrations of QS-molecules and QS-controlled bacterial traits (Jesaitis et al., 2003; Jensen et al., 2007, 2010). We have also shown recently that macrophage movement and engulfment of *P. aeruginosa* are paralleled by increased AQP9 expression and changes in its cellular distribution. Here, bacteria with a fully functioning AHL-dependent QS-system seem to elicit stronger responses than a *lasI-/rhlI*- mutant lacking C_4_-HSL and 3O-C_12_-HSL and thereby several virulence factors (Holm et al., 2015). It is, however, not clear enough whether *P. aeruginosa* QS-molecule 3O-C_12_-HSL itself impacts cell morphology and volume by changing AQP9 characteristics that may elicit danger signal and act as a chemoattractant for human macrophages.

To assess in greater detail of bacteria-host cell communication, we here investigated the effects of *P. aeruginosa* 3O-C_12_-HSL on cell morphology, area, volume and AQP9 characteristics of human primary macrophages, using quantitative PCR, immunoblotting, live 2D and 3D imaging, confocal, and nanoscale imaging.

## Materials and methods

### Ethics statement

The study was conducted in accordance with the Declaration of Helsinki. Human blood was collected and buffy coat was obtained by employees at the Blood Bank at Linköping University Hospital, Sweden. A written consent for research use of donated blood was obtained from all donors. Since blood donation is classified as negligible risk to the donors and since only anonymized samples were delivered to the researchers, the study did not require ethical approval according to paragraph 4 of the Swedish law (2003:460; http://www.lagboken.se/dokument/Lagar-och-forordningar/4060/Lag-2003_460-om-etikprovning-av-forskning-som-avser-manniskor?id=64991) on Ethical Conduct in Human Research.

### Isolation and culture of human primary monocytes

Human primary monocytes were isolated from healthy blood donor buffy coat. The latter was mixed with cold 0.9% NaCl (50/50), and leukocyte concentrate was obtained using Lymphoprep gradient (Axis Shield PoC AS, Oslo, Norway) after centrifugation for 40 min at 480 x g at room temperature (RT). The mononuclear cells were collected from the gradient, washed thrice in cold PBS, pH 7.3 containing heparin (0.1 μl/ ml, LEO Pharma Ballerup, Denmark), thrice in cold Krebs-Ringer Glucose buffer (KRG), pH 7.3 (120 mM NaCl, 8.3 mM KH_2_PO_4_, 4.9 mM KCl, 1.2 mM MgSO_4_, 10 mM glucose), and suspended in Dulbecco's Modified Eagle Medium (DMEM) supplemented with 25 mM HEPES, 100 U/ml penicillin and 100 μg/ ml streptomycin (Life Technologies, Grand Island, NY). The cells were seeded in culture flasks and left to adhere for 1.5–2 h at 37°C in 5% CO_2_ and 95% moisture, than the unbound cells were washed away with 37°C warm complete KRG (as above with addition of 1 mM CaCl_2_). The adherent cells were left to differentiate to macrophages in DMEM supplemented as above and with addition of 10% human serum and 80 μM L-glutamine (Life Technologies). After 7–8 days the cells were considered to be differentiated macrophages, according to their morphology and phagocytic properties to engulf two different prey, wild-type *P. aeruginosa* PAO1 (Holm et al., 2015), or heat-killed yeast *Saccharomyces cerevisiae* (Vikström et al., 2005) at a macrophage: prey ratio of 1:10. For experiments, mature macrophages were harvested with trypsin-EDTA (Life Technologies), washed, counted and seeded at concentration 10^6^ cells/well in 6-well plates for further qPCR and immunoblotting, or at 5 × 10^4^ on MatTek dishes (MatTek Corporation, Ashland, MA) for 2D and 3D imaging, or on glass coverslips (thickness 0.17 ± 0.01, 13 mm-diameter; Karl Hecht Assistent, Sondheim, Germany) for LSCM and STED imaging. Cells were left to adhere in DMEM as above for 2 h at 37°C in 5% CO_2_, and the experiments were conducted in DMEM supplemented as above without serum and L-glutamine.

### AHL synthesis

*N*-3-oxo-dodecanoyl-L-homoserine lactone C_16_H_27_NO_4_, MW 297.4 (3O-C_12_-HSL) was synthesized by Prof. Peter Konradsson and Lan Bui (Dept. of Organic Chemistry, Linköping University, Sweden) as previously described (Chhabra et al., 2003). These molecules are structurally and functionally identical to those obtained from *P. aeruginosa* cultures. The resulting 3O-C_12_-HSL was checked for identity and purity by HPLC, and its activity as a QS-molecule was confirmed by the bioassays described earlier (Surette and Bassler, 1998; Winson et al., 1998). For experiments, 3O-C_12_-HSL was dissolved in 100% dimethylsulfoxide (DMSO) as a stock solution and then further diluted in PBS, pH 7.3.

### Treatment with AHL and AQP inhibitors

Macrophages were treated with 3O-C_12_-HSL at 10 or 50 μM and further proceeded for live 2D and 3D imaging at 37°C in 5% CO_2_. For q-PCR, immunoblotting, LSCM and STED, cells were treated with 3O-C_12_-HSL at 10, 50, or 100 μM for 1, 4, or 24 h at 37°C in 5% CO_2_. As vehicle for 3O-C_12_-HSL, 0.02% DMSO was used. To examine the role of AQP9, 25 μM HTS13286 (Maybridge, Cornwall, UK), a specific peptide inhibitor of AQP9 (Jelen et al., 2011; Karlsson et al., 2013; Wacker et al., 2013), was added to the cells simultaneously with 3O-C_12_-HSL stimulation. As vehicle for HTS13286, 0.25% DMSO was used.

### Live 2D imaging

To examine cell morphology and area changes, time-lapse videos were recorded using the JULI bench top microscope (NanoEnTek Inc., Seol, Korea) inside the incubator at 37°C in 5% CO_2_. Image acquisition was performed at an interval of 1 min for 3 h. The images of the cells were analyzed using the ImageJ Fiji software (NIH, Bethesda, MD).

### Live 3D holographic imaging

To analyze optical cell volume changes, time-lapse videos were recorded using the HoloMonitor M4 (Phase Holographic Imaging PHI AB, Lund, Sweden) inside the incubator at 37°C in 5% CO_2_. Image acquisition was set at an interval of 30 s for 1 h. The images were analyzed using the Hstudio software (Phase Holographic Imaging PHI AB).

### LSCM and STED imaging

Samples were washed with complete KRG, fixed with 4% paraformaldehyde (Sigma Aldrich) for 20 min, blocked in 1% BSA and stained with rabbit anti-AQP9 antibodies (#ab84828-100, Abcam, UK). For classical laser scanning confocal microscopy (LSCM), the specimens were further labeled with Alexa Fluor 568-conjugated goat anti-rabbit antibodies (Life Technologies), diluted 1:1000 for 1 h at RT. For Stimulated Emission Depletion (STED) nanoscopy, Atto647N (STED) goat anti-rabbit antibodies (Active Motif, Carlsbad, CA) were added for labeling and incubated for 1 h at RT. Finally, coverslips were washed in PBS and mounted on glass microscope slides in ProLong Gold antifade reagent (Molecular Probes, Invitrogen). For LSCM, the specimens were examined through a 63 × oil immersion objective with NA 1.40 in a Zeiss Axio Observer Z1 fluorescence microscope with the Zeiss LSM 700 confocal system and Zeiss ZEN software (Carl Zeiss, Jena, Germany). STED nanoscale visualization allows capture and study subcellular architecture and dynamics at the nanoscale resolution that is 5–10 times higher (<20–40 nm), than with LSCM, and achieved by probing the sample with spatially modulated illumination (Blom and Brismar, 2014). Here, the specimens were examined in the Leica TCS SP8 STED 3X super-resolution microscope equipped with two continuous wave lasers at 592 and 660 nm and a pulsed laser at 775 nm and a 100×/1.4 oil immersion objective (Leica Microsystems, Mannheim, Germany). Fluorescence intensities and cell size for LSCM and STED images were measured and quantified using the ImageJ Fiji software (NIH, Bethesda, MD) and LAS AF software (Leica Microsystems, Mannheim, Germany). Deconvolution of the images was performed with the Huygens software (Scientific Volume Imaging, Hilversum, the Netherlands).

### Quantitative PCR (qPCR)

To determine differences in the expression of AQP9 in macrophages, qPCR were performed using GAPDH as reference gene which expression was not significantly influenced by treatment with 3O-C_12_-HSL at 10, 50, or 100 μM. The GeneJET RNA Purification Kit (#K0731, Thermo Scientific), High capacity RNA-to-cDNA Kit (random hexameres) and the Taqman Fast Universal PCR Master Mix (all from Applied Biosystems, Stockholm, Sweden) were used for extraction, reverse transcription and amplification, respectively according to the manufacturer's instructions. The amount of cDNA for the qPCR was set to 50 ng/well. The master mix of TaqMan Gene Expression Assay Mix (20X, 1 μl/sample, AQP9 Ref.Seq: NM_020980.3 Amplicon length: 55 Assay ID: Hs00175573 or GAPDH RefSeq: NM_002046.3 Probe Exon Location: 3 Amplicon Size: 122 Part No. 4333764T), TaqMan Fast Universal PCR Master Mix (2X) No AmpErase UNG (10 μl/sample) (all from Applied Biosystems) and RNase free water was placed in the MicroAmp Fast Optical 96-well reaction plate (Applied Biosystems) and cDNA was added to a total volume of 20 μl. All samples were run in triplets. The plate was sealed with Optical Adhesive cover (Applied Biosystems) and the PCR was run according to the 7500FAST programme; step 1 95°C 20 s, step 2 95°C 3 s, and step 3 60°C 30 s. Step 2 and 3 were repeated for 40 cycles. The results were analyzed using the 7300 System Software (Applied Biosystems). The Ct value was used to calculate fold-increase in AQP9 compared relative to reference gene GAPDH and the control.

### Total cell lysates, SDS-PAGE and immunoblotting

The samples were washed with PBS, pH 7.6 and lysed with ice-cold RIPA buffer (150 mM NaCl, 1% deoxycholic acid sodium salt, 1% NP-40, 0.1% SDS, 10 mM EDTA pH 8.0, 10 mM Tris pH 7.4 dissolved in PBS) supplemented with 25U nuclease (Thermo Scientific), 1 mM phenyl-methyl-sulfonyl-fluoride, 1 mM Na_3_VaO_4_, 25 mM NaF (Sigma), protein inhibitors Complete (Roche Diagnostics, Mannheim, Germany). Cell suspensions were homogenized through a 21-gauge needle and centrifuged at 18,000 g for 30 min at 4°C, and the supernatants were collected. The protein concentration in cell lysates was measured with the Bio-Rad D_*C*_ protein assay (Bio-Rad Laboratories, Hercules, CA). The samples were further diluted in Laemmli sample buffer at equal protein concentrations, heated for 5 min at 95°C and then subjected to electrophoresis. They were loaded on 4–12% SDS-polyacrylamide gels (Lonza, Rockland, ME), and after separation, proteins were electrophoretically transferred to a PVDF Immobilon-FL membrane (Millipore, Bedford, MA); the quality of the transfer was controlled by Ponceau S staining (Sigma Aldrich). Non-specific binding was blocked by 1-h incubation in 5% non-fat milk in PBS, pH 7.6 containing 0.18% Tween 20 at RT. The membranes were then incubated with rabbit anti-AQP9 antibodies (#ab85910 Abcam) or anti-GAPDH antibodies (Millipore, Temecula, CA) diluted 1:2000 in blocking buffer overnight at 4°C. After washing, they were treated for 1 h at RT with IRDye 800CW goat anti-rabbit or IRDye 680CW goat anti-mouse antibodies (LI-COR Biosciences, Cambridge, UK), diluted 1:10,000 and washed extensively. The signals were detected and the density ratio was quantified using Odyssey CLx and the Image Studio software (LI-COR).

### Statistical analyses

Data on optical volume are presented as a percent of change, the values represent moving average of the means ± SE, and significant differences were analyzed by two-way ANOVA and Bonferroni's post-test. Data in all other graphs are presented as mean ± SE. Results from live 2D imaging were analyzed using Student's *t*-test. The qPCR experiments were analyzed by two-way ANOVA and Bonferroni's post-test. Data from immunoblotting were analyzed by one-way ANOVA-Friedman and Dunn's multiple comparison test. The whole cell fluorescent intensity measurements were analyzed by one-way ANOVA and Dunnett's multiple comparison test and approximated cell area and length in AQP9-stained macrophages were analyzed using one-way ANOVA. The numbers (*n*) are specified in the figure legends. *P* < 0.05, < 0.01, and < 0.001 were considered significant.

## Results

### 3O-C_12_-HSL increases the cell area and protrusive activity of macrophages

**Figure 1:**
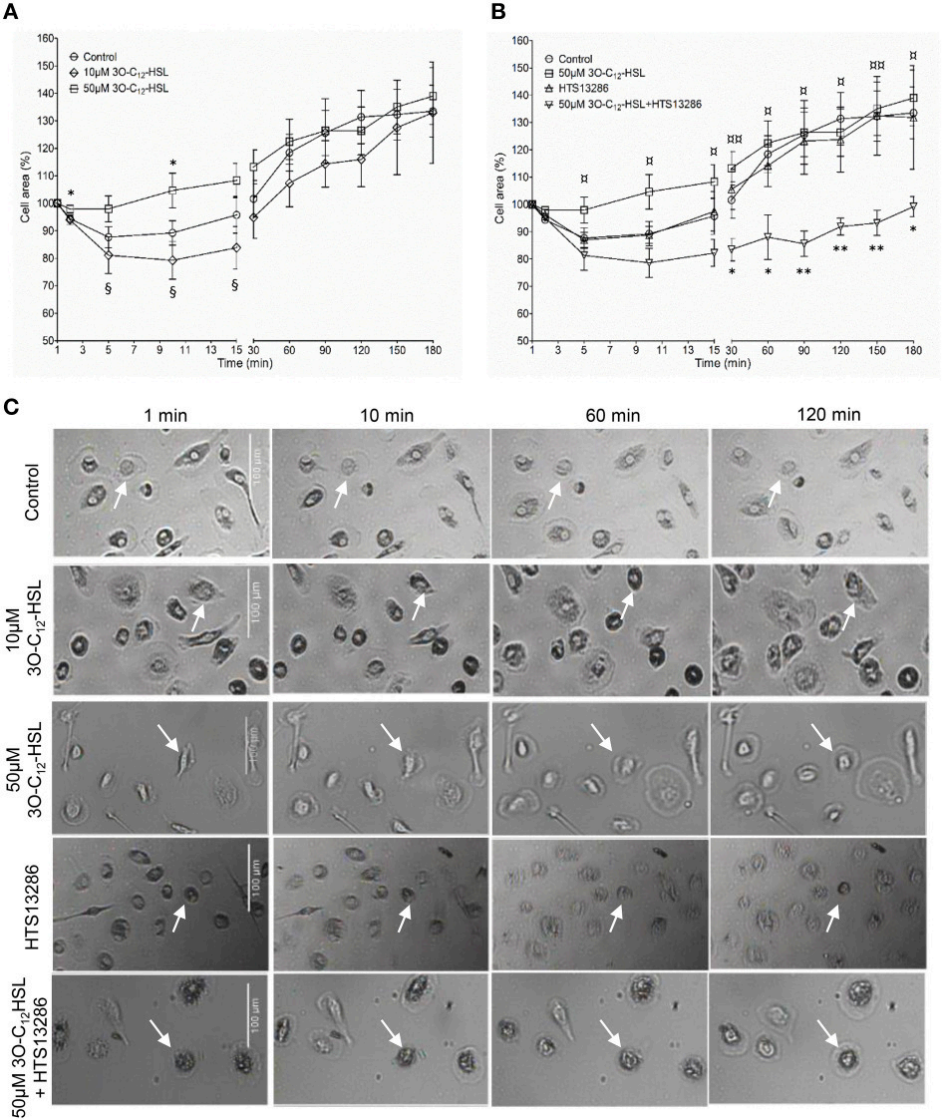
**Live 2D imaging of cell area and protrusive activity in 3O-C_**12**_-HSL-stimulated macrophages. (A)** Macrophages were treated either with 0.02% DMSO (Control), or 10 and 50 μM 3O-C_12_-HSL. **(B)** Cells were treated with 50 μM 3O-C_12_-HSL, 25 μM HTS13286, or simultaneously with both 50 μM 3O-C_12_-HSL and 25 μM HTS13286, or 0.27% DMSO (not shown) or 0.02% DMSO as a vehicle control (Control). Control groups and 50 μM 3O-C_12_-HSL groups were the same in **(A)** and **(B)**. A time-lapse Videos S1–S5 were recorded using JULI and cell area was quantified and shown as percent (%) change relative to the initial area. Values are the mean ± SE (*n* = 8 for Control and 50 μM 3O-C_12_-HSL; *n* = 3 for 10 μM 3O-C_12_-HSL; *n* = 4 for 25 μM HTS13286; and 50 μM 3O-C_12_-HSL+25 μM HTS13286) based on at least 20 cells for each condition and experiment. Significant differences were analyzed by one-tailed, un-paired Student's *t*-test and are indicated with ^*^ or ^**^ when *P* < 0.05 or *P* < 0.01 compared to control, with ¤or ¤¤when *P* < 0.05 or *P* < 0.01 comparing 50 μM 3O-C_12_-HSL and 50 μM 3O-C_12_-HSL+HTS13286, and with § when *P* < 0.05 comparing 10 and 50 μM 3O-C_12_-HSL. **(C)** Representative images from typical time-lapse Videos S1–S5 recorded with 2D imaging. White arrows point on macrophages with typical outcomes for cell areas. Bar 100 μm.

*P. aeruginosa* AHL signal molecules may stimulate both leukocyte chemotaxis and phagocytosis (Vikström et al., 2005; Zimmermann et al., 2006; Wagner et al., 2007; Karlsson et al., 2012), which require quick changes in cell size and morphology putatively driven by fluxes of water across the membrane through AQP. Therefore, we first investigated whether *P. aeruginosa* 3O-C_12_-HSL affected cell area, the rate of cell polarization and membranous protrusion activity using 2D live imaging that provides continuous monitoring over the time (Figure 1 and Figure S1). Generally, we noticed that cell areas in all groups including control were initially reduced and later increased reflecting the classical cycle of cell shape fluctuations, i.e., retraction, spreading and extension during macrophage rest and further spontaneous or stimulated movements (Figure 1). We observed that treatment with 50 μM 3O-C_12_-HSL, in contrast to 10 μM and the control, resulted in increased cell area in macrophages (Figure 1A). As shown in individual experiments in Figure S1 there was a clear increase in the relative cell area by 50 μM 3O-C_12_-HSL in macrophages from Donor I, II, IV, V, and VII compared to the control. In cells isolated from the other donors we observed a decreased (Donor III), modest (Donor VIII), or small (Donor VI) cell area changes after treatment with 50 μM 3O-C_12_-HSL. Time-lapse Videos S1–S5 with 2D live imaging show typical outcomes for macrophage cell area, motility and protrusive activity after 3O-C_12_-HSL-stimulation over 180 min. In Figure 1C, the images were obtained from a representative videos. Cell tracing data demonstrated that control macrophages maintained a similar pattern of morphology characteristics over the time, i.e., cell polarity, rounding, and development of distinct lamellipodia (Table 1). In contrast, macrophages treated with 10 or 50 μM 3O-C_12_-HSL revealed short-term cell shape fluctuations between polarity and rounding and also typical protrusion events (Table 1, Table S1), which are central in cell morphogenesis, behavior and cell locomotion. Next, we investigated whether blocking of water fluxes through AQP9 affected the morphology of macrophages by treating the cells with 25 μM HTS13286 alone or together with the 3O-C_12_-HSL-stimulation. Thus, blocking of AQP9 alone resulted in a slight cell rounding with significant effect at 15 min (Table 1); cell area was near to control (Figure 1B). Further, we detected that inhibition of AQP9 prevented cell area increases (Figure 1B), polarization and protrusions fluctuations in 3O-C_12_-HSL-treated cells (Table 1, Table S1). In the individual experiments shown in the lower 4 panels in Figure S1, we observed that the area of the cells were smaller when 3O-C_12_-HSL were combined with the HTS13286 inhibitor (orange triangles), than for 3O-C_12_-HSL alone (red squares) for all donors at all time points. Together, these live imaging data demonstrate that *P. aeruginosa* 3O-C_12_-HSL signal molecules provoke quick expansion of cell area and changes in morphology in human macrophages and that these processes are driven by fluxes of water through AQP9.

**Table 1:** **Morphological changes in 3O-C_**12**_-HSL-stimulated macrophages identified by 2D live imaging**.

**Time (min)**	**Control**	**10 μM 3O-C_12_-HSL**	**50 μM 3O-C_12_-HSL**	**HTS13268**	**HTS13268 and 50 μM 3O-C_12_-HSL**
**POLARIZED MACROPHAGES (% OF TOTAL CELLS)**					
1	44±7	53±7	54±5	33±5	34±10^¤^
10	41±7	35±3^*^^#^	51±5	25±5	31±11^¤^
15	44±6	47±4	46±4	25±5^*^	30±10^*^
30	40±7	37±2^*^^#^	41±6^§^	30±6	29±10
90	43±7	35±3^*^^#^	39±4^#^^§^	33±6	20±5^*^^¤^
180	41±6	52±12	38±5^#^	28±8	21±5^*^^¤^
**MACROPHAGES WITH ONE OR MORE DISTINCT LAMELLIPODIA (% OF TOTAL CELLS)**					
1	43±4	50±5	49±7	36±3	45±8
10	38±5	22±11^#^	41±4^§^	29±8	38±12
15	35±4	22±12^#^	46±4^*^^§^	28±8	30±13
30	38±5	28±2^#^^#^	47±5^§^	31±9	28±7^¤^
90	44±6	37±9	41±5	34±7	28±6
180	45±6	42±15	43±7	46±13	30±8

Significant differences were analyzed by one-tailed unpaired Student's t-test and are indicated with:

* compared to control at the same time point;

# within the group (eg. 50 μM 3O-C_12_-HSL) compared to the 1 min-value in the group;

¤ 50 μM 3O-C_12_-HSL vs. 50 μM 3O-C_12_-HSL+HTS13286;

§ *10 μM 3O-C_12_-HSL vs. 50 μM 3O-C_12_-HSL*.

### 3O-C_12_-HSL enhances cell volume in macrophages

**Figure 2:**
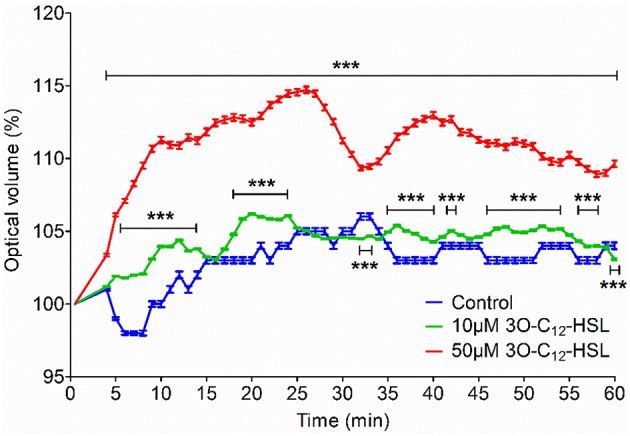
**Volume changes in 3O-C_**12**_-HSL-stimulated macrophages analyzed by holographic 3D imaging**. Macrophages were treated with 10 and 50 μM 3O-C_12_-HSL, or 0.02% DMSO as a vehicle control (Control) and a time-lapse video was recorded using the HoloMonitor M4. Quantification of optical volume shown as a percent (%) of change. Values represent the moving average of the mean ± SE based on 4 independent experiments from 4 different donors and 120–300 cells for each condition. Significant differences were analyzed by two-way ANOVA and Bonferroni's post-test and are indicated with ^***^ when *P* < 0.001 compared to control.

Since 3O-C_12_-HSL-treatment caused increased cell area in the macrophages, parallel changes in cell volume driven by water transport might occur. To test this, we used live 3D holographic imaging and subsequent quantification of changes in the optical volume of macrophages over time (Figure 2). After treatment with 10 μM 3O-C_12_-HSL, we noticed a modest increase in the optical volume in macrophages compared to control cells; this was significant during several time periods, i.e., around 10, 20, 40, and 50 min. Treatment with 50 μM 3O-C_12_-HSL resulted in a more pronounced increase in the optical volume in the macrophages by 9.0%, compared to the control, with significance during the whole time course (Figure 2). Thus, 3D holographic, live-cell visualization data corroborated that *P. aeruginosa* 3O-C_12_-HSL enhanced the volume of macrophages, being consistent with our results on increased cell area and protrusive activity from 2D live imaging (Figure 1).

### 3O-C_12_-HSL increases the expression of AQP9 in macrophages

**Figure 3:**
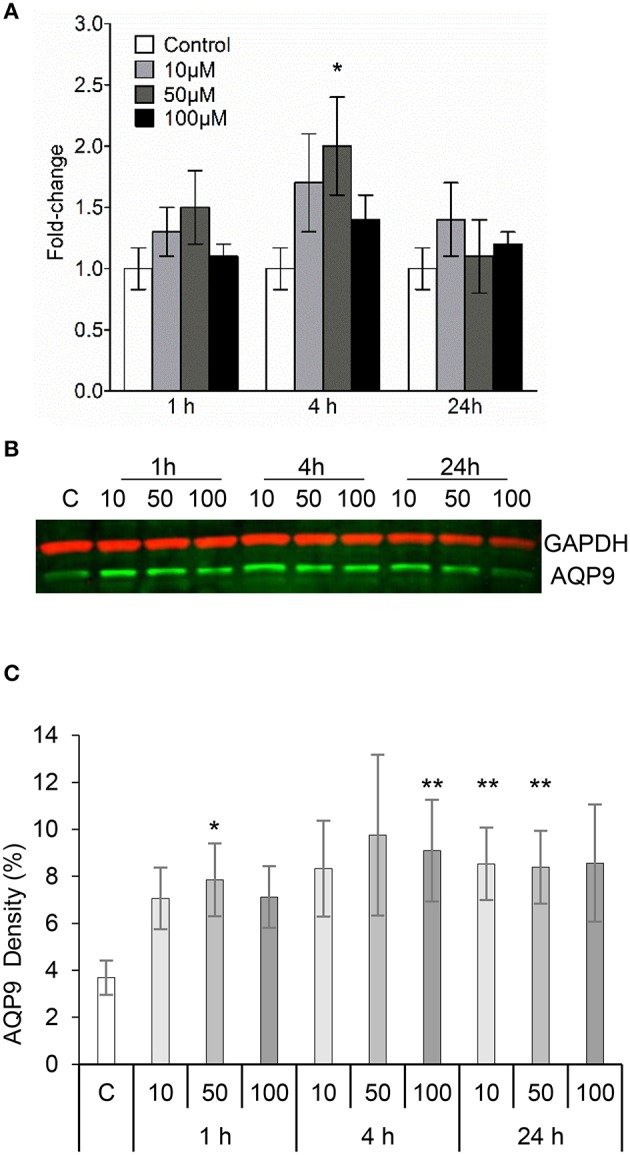
**3O-C_**12**_-HSL increases the expression of AQP9 in macrophages. (A)** Changes in AQP9 mRNA levels of macrophages. Cells were treated either with diluent (Control) or with 10, 50, and 100 μM 3O-C_12_-HSL for 1, 4, and 24 h, and qPCR was performed using GAPDH as a reference gene. Two-way ANOVA suggested that the concentration but not the different times of observation primarily affected the expression rate. Significant difference is indicated with ^*^ when *P* < 0.05, as analyzed by Bonferroni's post-test (*n* = 6). **(B)** Cells were treated either with diluent as a control **(C)** or with 10, 50, and 100 μM 3O-C_12_-HSL for 1, 4, or 24 h. Whole cell lysates were analyzed with immunoblotting for AQP9 and GAPDH for loading control. The blot is representative of six independent experiments from six different donors. **(C)** Quantification of blots. AQP9 levels normalized to the GAPDH control are indicated as percent (%). Values are the mean ± SE (*n* = 6). Significant differences are indicated with ^*^ when *P* < 0.05 and ^**^ when *P* < 0.01, as analyzed by one-way ANOVA-Friedman and Dunn's multiple comparison test.

Human leukocyte adaptation to different environmental constraints requires changes in volume, presumably associated with water fluxes over the plasma membrane through the predominant AQP9 (Loitto et al., 2002; Karlsson et al., 2011, 2013). We have recently shown that infections with *P. aeruginosa* do increase the AQP9 expression in macrophages, and that bacteria with a fully functional QS-system provoke a larger increase compared to a mutant lacking 3O-C_12_-HSL and C_4_-HSL (Holm et al., 2015). Because macrophages changed their cell area and volume in response to 3O-C_12_-HSL, we decided to further investigate whether the expression of AQP9 at mRNA and protein levels were affected. This was analyzed by qPCR (Figure 3A) and immunoblotting (Figures 3B,C) for the expression of AQP9 with GAPDH as a control. In general, after treatment of macrophages with 10, 50, and 100 μM 3O-C_12_-HSL for 1, 4, and 24 h, the mRNA levels were elevated compared to the dilution control (Figure 3A). Moreover, 4 h-treatment with 50 μM 3O-C_12_-HSL caused a significantly increased and almost 2-fold greater AQP9 mRNA expression. Next, immunoblotting for AQP9 and GAPDH revealed generally higher levels of AQP9 in macrophages after 3O-C_12_-HSL-stimulation (Figures 3B,C). Furthermore, a significant increase in AQP9 expression level was detected in macrophages treated with i.e., 50 μM for 1 h, 100 μM for 4 h, 10 μM for 24 h, and 50 μM for 24 h. Taken together, this suggests that 3O-C_12_-HSL does increase the expression of AQP9 at both protein and mRNA levels in human macrophages.

### The cellular redistribution of AQP9 and morphology of macrophages are changed in response to 3O-C_12_-HSL

**Figure 4:**
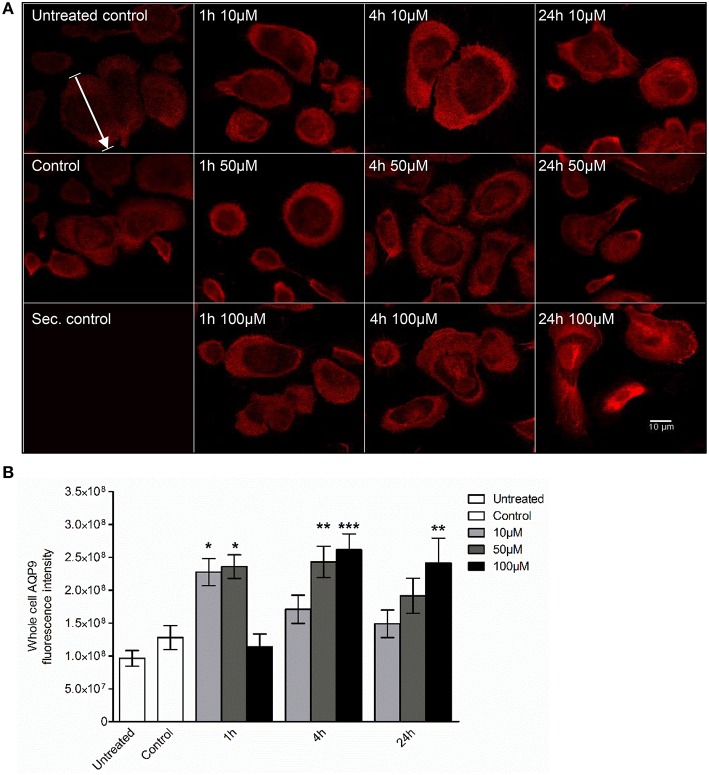
**Visualization of AQP9 cellular distribution in 3O-C_**12**_-HSL-stimulated macrophages. (A)** Macrophages were either untreated (Untreated control), or treated with diluent (Control), or stimulated with 10, 50, and 100 μM 3O-C_12_-HSL for 1, 4, or 24 h, stained for AQP9 (red) and analyzed by LSCM. Staining with secondary antibody (Sec. control) as a control. The data are from one of six independent experiments. Bar 10 μm. **(B)** Quantification of AQP9 immunofluorescence intensity measured as a total integrated intensity density of whole macrophage area. Columns represent the means ± SE. Significant differences are indicated with ^*^ when *P* < 0.05, ^**^ when *P* < 0.01, and ^***^ when *P* < 0.001, as analyzed by one-way ANOVA and Dunnett's multiple comparison test. Data from at least four different experiments performed on separate days from six different donors were used, and at least 100 cells in total per condition were analyzed.

**Figure 5:**
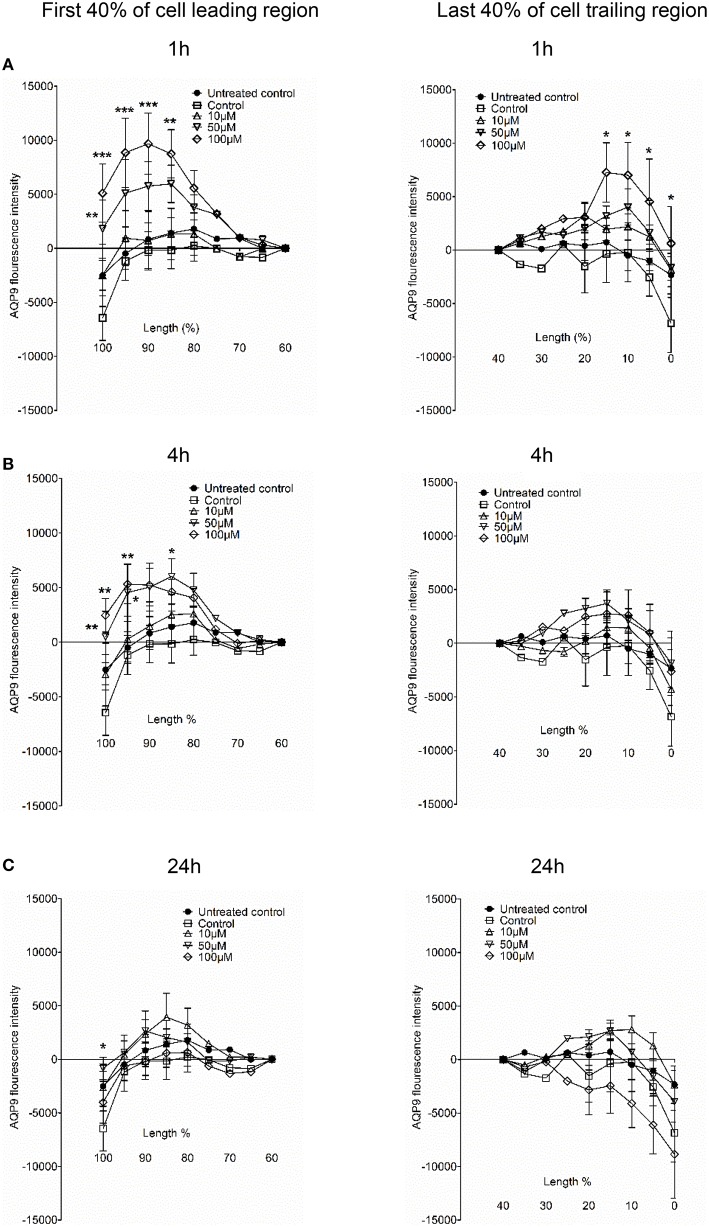
**AQP9 intensity profiles across the leading and trailing regions in macrophages**. The experiment, measurements and the quantifications of AQP9 intensity profiles over the cell in the direction of polarization were performed as shown by white arrow in Figure 4A. AQP9 intensity difference in the first 40% of cell leading region (left row) and last 40% of cell trailing region (right row) in macrophages after 3O-C_12_-HSL-treatments for 1, 4, and 24 h are shown in **(A), (B)**, and **(C)**, respectively. Values are the mean ± SE. Significant differences are indicated with ^*^ when *P* < 0.05, ^**^ when *P* < 0.01, and ^***^ when *P* < 0.001, as analyzed by one-way ANOVA. Data are from at least 4 different experiments performed on separate days from six different donors, and at least 100 cells in total per condition were analyzed.

**Figure 6:**
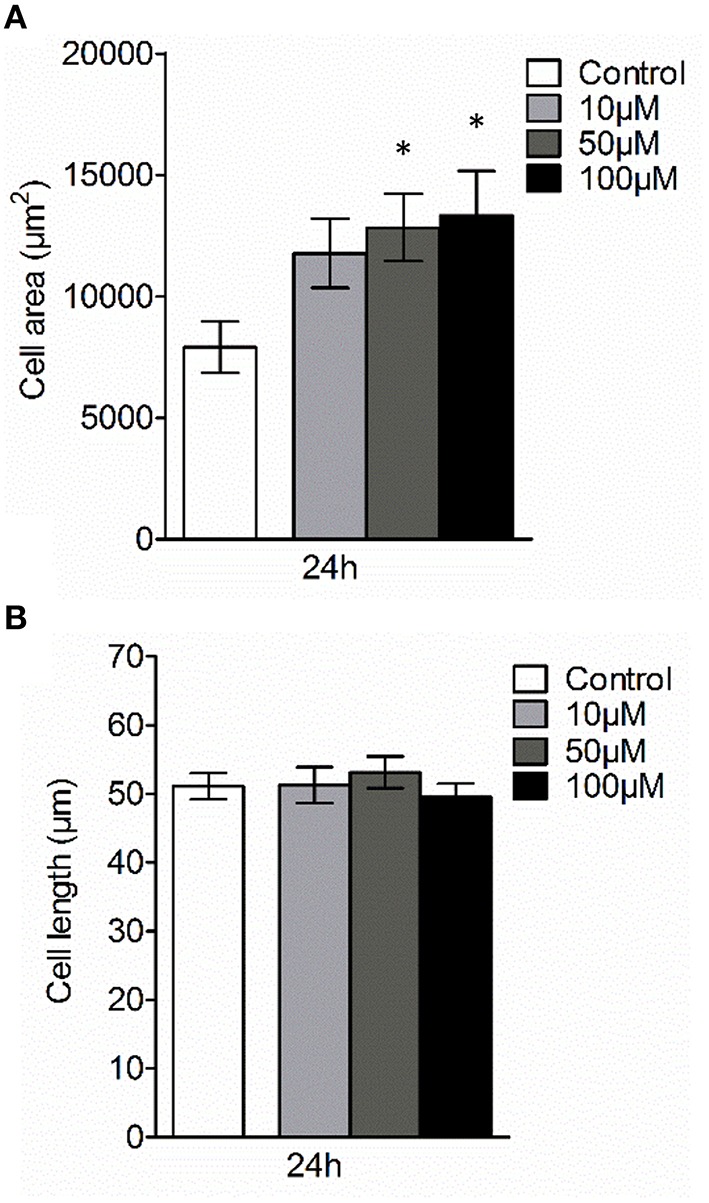
**Effect of 3O-C_**12**_-HSL on AQP9-related cell size of macrophages**. Cell were either treated with diluent 0.02% DMSO (Control) for 24 h, or stimulated with 10, 50, and 100 μM 3O-C_12_-HSL for 24 h, stained for AQP9, analyzed by LSCM as shown in Figure 4A. **(A)** Quantification of AQP9-related cell area. **(B)** Quantification of the approximate cell length of AQP9-stained macrophages (measured as indicated by white arrow in Figure 4A in the direction of polarization). Columns represent the means ± SE. Data from at least four different experiments performed on separate days from six different donors, and at least 100 cells in total per condition. Significant differences are indicated with ^*^ when *P* < 0.05, as analyzed by one-way ANOVA and Dunnett's multiple comparison test.

Next, we examined whether 3O-C_12_-HSL could affect the cellular distribution of AQP9 using laser scanning confocal microscopy (LSCM) and quantification of fluorescence intensities in images (Figure 4). Macrophages were incubated with diluent control or 10, 50, and 100 μM 3O-C_12_-HSL for 1, 4, and 24 h. The control cells displayed very low levels of AQP9, distributed in cytoplasmic area. However, after treatment with 3O-C_12_-HSL, we observed that whole cell AQP9 fluorescence intensity was significantly increased for five doses and time points (Figures 4A,B). In parallel, we also measured AQP9 fluorescence intensity over the macrophages in the direction of their polarization, as shown by the white arrow in Figure 4A. This revealed a pronounced and significant redistribution of AQP9 to the leading and trailing regions in 3O-C_12_-HSL-stimulated cells compared to controls, which had more evenly distributed AQP profiles (Figure 5 and Figure S2). When turning to the measurements of AQP9-related cell area in macrophages using LSCM images, we found significant increase after 24-h stimulation with 50 μM 3O-C_12_-HSL compared to the control (Figure 6A). However, some inconsistency between these results and data from cell area quantification from 2D imaging (Figure 1A) could depend on different approaches used to measure cell areas: confocal imaging of AQP9-stained cell with higher resolution over longer time vs. imaging of living cells with lower resolution over shorter time. The length of the AQP9-stained cells, as measured in the direction of polarization (as shown in Figure 4A) was mainly unaltered compared to the control (Figure 6B). These data suggest that *P. aeruginosa* 3O-C_12_-HSL induces cellular redistribution of AQP9 in macrophages and in parallel increases the cell area.

### 3O-C_12_-HSL induces changes in AQP9 dynamic and architecture at nanoscale resolution

**Figure 7:**
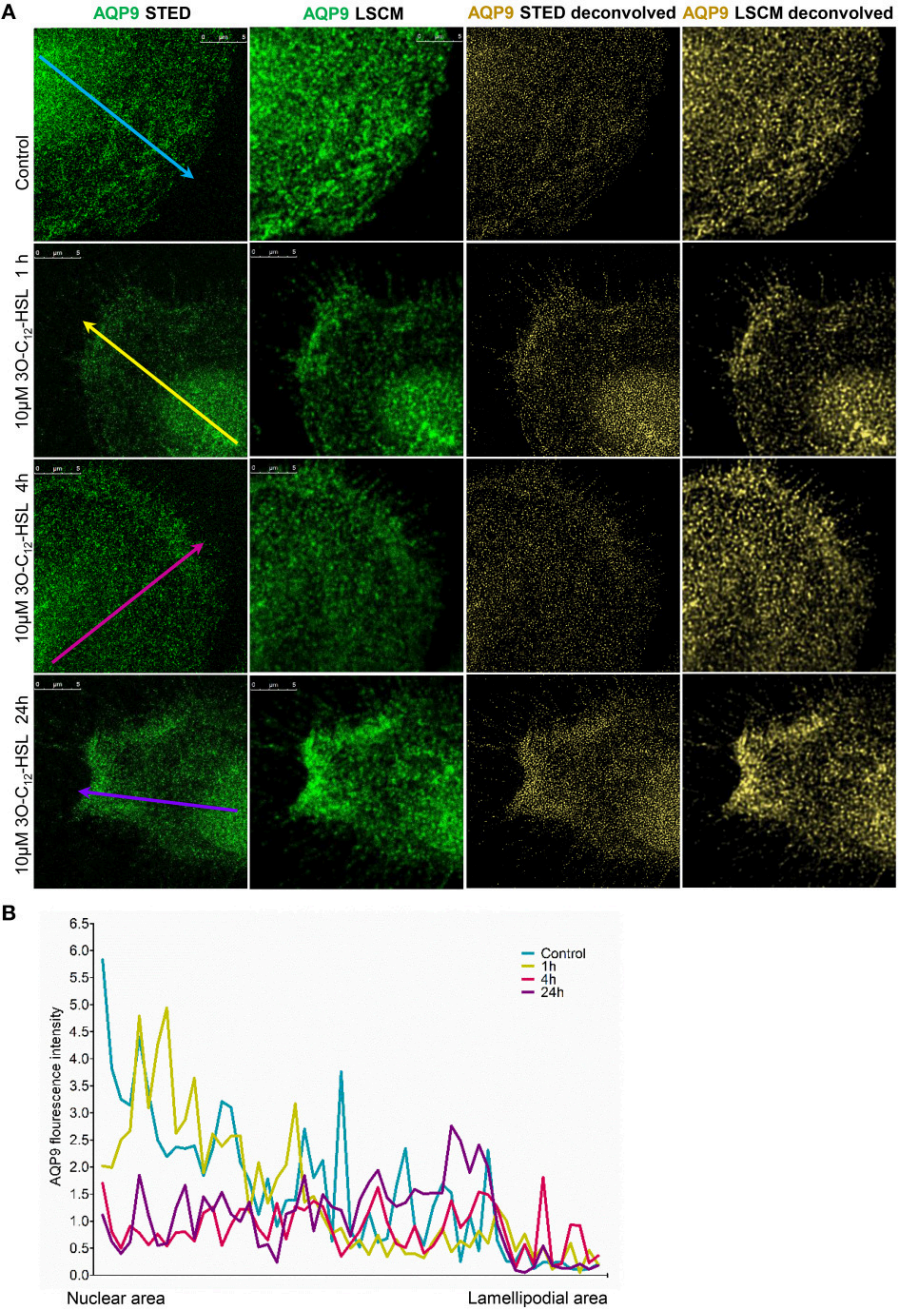
**Nanoscale imaging of AQP9 architecture in 3O-C_**12**_-HSL-stimulated macrophages. (A)** Macrophages were stimulated with 10μM 3O-C_12_-HSL for 1, 4 or 24 h, or 0.02% DMSO as a vehicle control (Control), stained for AQP9 (green) and analyzed with STED nanoscopy. The two left panels show the images after Huygens deconvolution (gold) with improved visualization quality. Bar 5 μm. **(B)** AQP9 fluorescence intensity profiles over the cells, measured as indicated by colored arrows in **(A)** in the direction from nuclear to lamellipodial area on the representative images.

**Figure 8:**
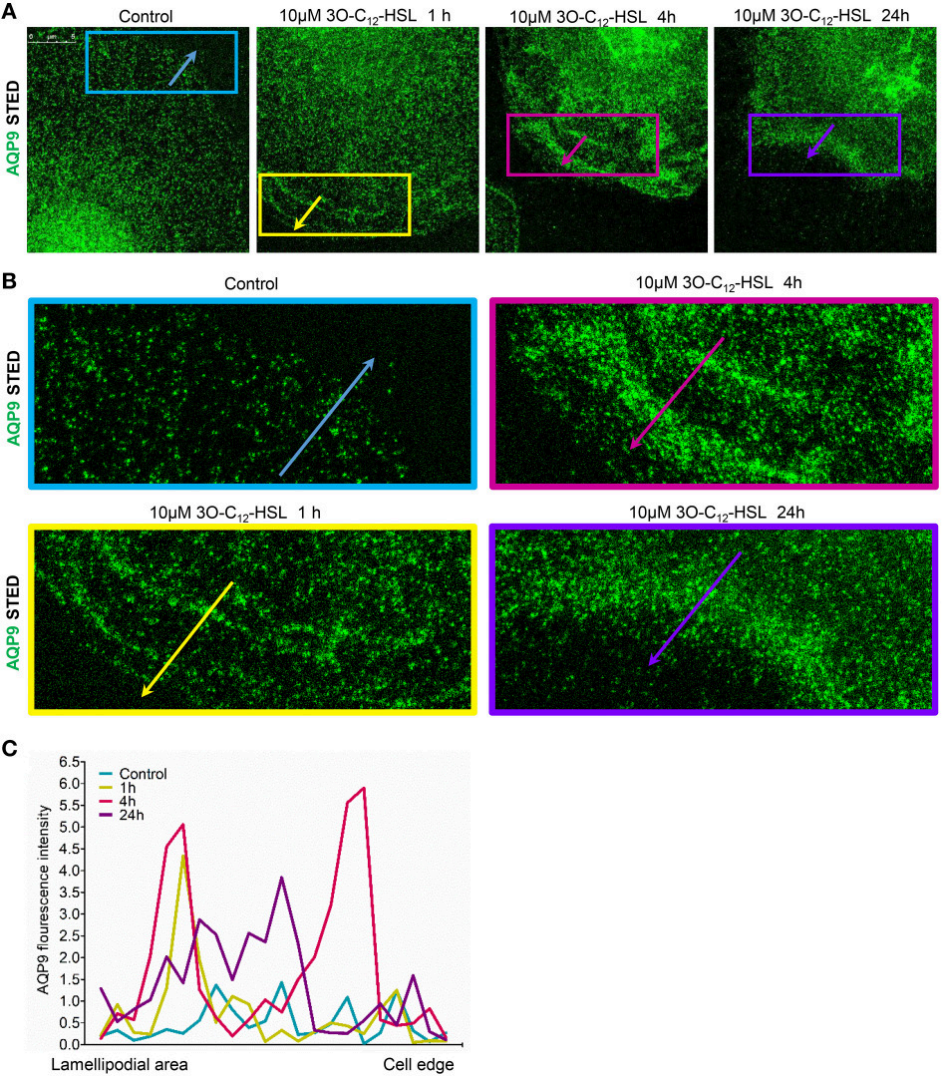
**Nanoscale visualization of AQP9 dynamic in lamellipodial area in 3O-C_**12**_-HSL stimulated macrophages**. Macrophages were stimulated with 10 μM 3O-C_12_-HSL for 1, 4, or 24 h, or 0.02% DMSO as a vehicle control (Control), stained for AQP9 (green) and analyzed with STED nanoscopy. **(A)** Main panels, bar 5 μm. **(B)** Inserts, image size is 15.5 × 6.2 μm. **(C)** AQP9 fluorescence intensity profiles measured as indicated by colored arrows in **(A, B)** in the direction from lamellipodial area to the edge of macrophages on the representative images.

To further study the details of redistribution of AQP9 in macrophages, we used stimulated emission depletion nanoscopy (STED) which allows nanoscale visualization at a resolution of around 20–40 nm (Figures 7, 8). Because macrophages changed their optical volume (Figure 2), AQP9 levels (Figure 3), and cellular distribution (Figures 4, 5) in response to 10 μM 3O-C_12_-HSL, we decided to further investigate whether low concentration of 3O-C_12_-HSL affected nanoscale AQP9 dynamics and architecture. When cells were treated with diluent or 10 μM 3O-C_12_-HSL for 1, 4, and 24 h, we observed more distinct AQP9 localization at peripheral regions in vicinity of leading- and trailing- edges and also in protruding structures in macrophages. By applying Huygens deconvolution we could further improve the visualization quality of both STED and confocal images (Figure 7A). Just like with LSCM, the measurements of AQP9 fluorescence intensity over the cells, in the direction from nuclei to membrane area, revealed distinct profiles and AQP9 accumulation in the edge regions in 3O-C_12_-HSL-stimulated macrophages compared to the control (Figure 7B). Within the lamellipodial area of the 3O-C_12_-HSL-stimulated cells, we were surprised to find distinct longitudinal wave patterns of AQP9 intensity, placed perpendicularly to the direction of suggested cell movement, thus, identifying the regions of AQP9 compression and rarefaction (Figures 8A–C). Together, *P. aeruginosa* 3O-C_12_-HSL caused distinct changes in the dynamic and structural nanoscale organization of AQP9 in moving human macrophages.

## Discussion

In leukocytes, cell volume and shape changes are strongly dependent on the transport of water through AQP, which is also critical for their motility and phagocytosis in physiological conditions and also in infection and inflammation (Lang et al., 1998; Hoffmann et al., 2009). The effectiveness of innate immune cells to fight bacteria and, hereby resolve the early stages of infection and inflammation depends on both host defense capacity and microbe ability to accomplish collective activities through its QS-system, i.e., communication, production of biofilms and virulence factors. Here, using 2D and 3D imaging, we have demonstrated that *P. aeruginosa* QS-signal molecules, like 3O-C_12_-HSL enhance cell volume, area and protrusive activity in human macrophages and that these processes are likely driven by fluxes of water across membrane through AQP9 (Figures 1, 2 and Table 1). Sensing bacterial QS molecules and responding by cell swelling facilitated via AQP9 could be a danger signal and a way for the macrophage to prepare for migration to the site of infection. Formation of membranous protrusions, like blebs, filopodia, and lamellipodia, are important for cell motility in a 3D environment (Paluch and Raz, 2013) and AQP9 have been suggested to play a critical role in their formation. AQP9 mediates the influx of water and ions across membrane and then creates an enhanced local hydrostatic pressure, which forces the membrane to extend and allows a polymerizing actin cytoskeleton to fill the protrusion (Karlsson et al., 2013). Through this mechanism macrophages can create numerous protrusions, move to and phagocyte bacteria. Indeed, human macrophages mount more effective phagocytosis of *P. aeruginosa* harboring a fully functional QS system, paralleled with increased expression and distribution of AQP9 and also distinct changes in cell size and morphology. In fact, bacteria with a working 3O-C_12_-HSL-dependent QS-system and thereby several virulence factors contributed more to this process (Holm et al., 2015). Here, we have further clarified that *P. aeruginosa* QS-molecule 3O-C_12_-HSL impacts macrophage morphology and volume by changing its AQP9 characteristic.

As shown in Figure S1, there were eight donors, and therefore eight individuals, whose cells could behave distinctly by being genetically and epigenetically different and in various stages of activation and preparedness to respond to stimuli. The latter can occur before or during isolation of the cells, or when they attach and spread on substratum, albeit the same protocol is used. That is also why some donor cells may respond to stimuli by retraction rather than expansion, resulting in an apparently smaller cell area, which of course makes the interpretations more complex. Cells attaching and periodically moving over surfaces, i.e., expanding, retracting, rounding up, and expanding again are normal and expected behaviors. Thus, we could anticipate to see varying responses over time. This is also why not only the cell area but also the cell volume was assessed, using holographic imaging (Figure 2).

It is remarkable, that leukocyte can sense QS signals of AHL nature, affecting for instance migration (Zimmermann et al., 2006; Karlsson et al., 2012) and phagocytosis of a prey (Vikström et al., 2005; Wagner et al., 2007). An AHL with long and middle-chain fatty acid, e.g., 3O-C_12_-HSL and 3O-C_10_-HSL, does induce the migration and phagocytosis in a dose-dependent manner, which puts it in a position among potent chemoattractants, such as cytokines, platelet activation factor, and leukotriene B. Lung tissues from *P. aeruginosa*-colonized patients contains a great amount of infiltrated leukocytes and enhanced concentrations of cytokines, GM-SCF and TNF-α (Ulrich et al., 2010), and sputum from such patients and biofilms growing in the lung has detectable levels of QS molecules (Charlton et al., 2000; Singh et al., 2000; Erickson et al., 2002). Thus, C_4_-HSL and 3O-C_12_-HSL have been identified in the sputum from bacteria-infected cystic fibrosis patients at nM-concentrations (Erickson et al., 2002), which is surprisingly low, but could depend on the techniques used to collect and measure QS molecules in biological samples (Erickson et al., 2002). However, notably higher 3O-C_12_-HSL concentrations, at μM-range, were detected with LC-MS/MS when *P. aeruginosa* was cultivated planktonically *in vitro* (Ortori et al., 2011). In bacterial biofilms grown *in vitro*, 3O-C_12_-HSL molecules were accumulated at very high levels, yielding up to 300–600 μM (Charlton et al., 2000).

3O-C_12_-HSL has been shown to increase IL-8 production in bronchial epithelial cells and fibroblasts, leading to massive infiltration of leukocytes into the tissues (Smith et al., 2001). In line with this, swelling of epithelial cells during wound-induced low osmotic pressure lead to the production of chemoattractants and infiltration of leukocytes, indicating a role for AQP also in this process (Enyedi et al., 2013). Swelling and volume changes in macrophages driven by AQP have been proposed to activate the NLRP3 inflammasome, and thereby initiate the synthesis of the pro-inflammatory cytokine IL-1β (Compan et al., 2012). Bacterial 3O-C_12_-HSL signals do also upregulate the expression of IL-1β and IL-1α (Smith et al., 2002), and downregulate the production of another pro-inflammatory TNF-α (Telford et al., 1998) and anti-inflammatory IL-10 (Glucksam-Galnoy et al., 2013) in macrophages. 3O-C_12_-HSL-induced expression of destructive pro-inflammatory cytokines and enzymes is controlled through NF-κB pathway (Telford et al., 1998; Smith et al., 2001) and together with a variety of bacterial traits, may manipulate leukocytes apoptosis in the site of infection. However, 3O-C_12_-HSL has been demonstrated to cause apoptotic and cytotoxic effects only on cancer cells *in vitro* (Tateda et al., 2003) and on tumor growth in animal studies *in vivo* (Zhao et al., 2016). Human primary macrophages, as used in this study and earlier (Vikström et al., 2005; Holm et al., 2015), did not revealed any apoptotic-like changes after 3O-C_12_-HSL-treatment as observed by live imaging (Figure 1 and Videos S1–S5). Bacteria, using sophisticated QS communication, can respond collectively to the presence of leukocytes by increasing the production of extracellular virulence factors and also rhamnolipids important for biofilm development (Jensen et al., 2007; Alhede et al., 2009). These factors help bacteria colonize and destroy host cells and tissues resulting in a more severe outcome of an infection and the development of disease. Altogether, these illustrate the complex array of players in infection and inflammation events, where on the one hand QS-communication takes a critical impact in pathogenesis of bacteria, and on the other hand AQP takes its new role in immune and epithelial cell functions.

Here, we decided to further investigate the role of AQP9 during bacteria-macrophage communication via 3O-C_12_-HSL in greater detail using different technologies, including immunoblotting, qPCR, confocal microscopy and super-resolution nanoscopy. Our data led to a coherent picture of how 3O-C_12_-HSL contribute to different AQP characteristics in human macrophages (Figures 3–8).

First, using qPCR and immunoblotting, we demonstrated that 3O-C_12_-HSL causes increased expression of AQP9 at both mRNA and protein levels (Figure 3). These data provide a straightforward explanation for the increased cell volume (Figure 2), area and protrusive activity (Figure 1 and Table 1) of macrophages induced by 3O-C_12_-HSL. This is because AQP mediate transport of fluid across the membrane and are involved in cell volume regulation, cell movement, organelle physiology, metabolism and signal transduction (Verkman, 2005). The observation of a strict correlation between increased gene and protein expression for AQP9 is worth to notice, since in many cases this may not been seen due to the control at many levels, including transcriptional and translational regulation (Vogel and Marcotte, 2012). During QS communication, increased cell volume, area and protrusive activity of macrophages driven by AQP9-mediated transport of fluids may further regulate different cell processes and behaviors such as cell movement, phagocytosis, signal transduction, and inflammatory response.

Then, using classical immunofluorescence staining, confocal microscopy, analysis and quantification of images, we found that 3O-C_12_-HSL caused significantly enhanced whole cell AQP9 fluorescent intensity (Figures 4A,B), paralleled with increased cell area (Figure 6A) in the macrophages. These data are clearly in line with the increased expression of AQP9 at both mRNA and protein levels detected in 3O-C_12_-HSL-stimulated macrophages (Figure 3). Moreover, this was consistent with what we observed in our 2D and 3D live-cell experiments: 3O-C_12_-HSL enhances cell volume, area and protrusive activity in human macrophages and these processes are driven by fluxes of water across membrane through AQP9 (Figures 1, 2 and Table 1). Further analyses of the confocal images and quantification of AQP9 intensity profile over cell provided a picture of how AQP9 redistributes in cytoplasmic area in macrophages sensing 3O-C_12_-HSL signals. Thus, AQP9 relocalized to the leading and trailing regions in moving macrophages (Figures 4, 5), into the first and the last 40% of cell length. These elevated levels of AQP9 in the plasma membrane regions could drive more intensive influx of water and ions across membrane, which creates an enhanced local pressure and forces the membrane to extend and allow cytoskeleton to fill the protrusion. Using such numerous protrusions, macrophages can move to the site of infection and phagocyte bacteria.

Finally, STED microscopy allowing nanoscale resolution provided a more detailed picture of AQP9 dynamics and fine architecture within the lamellipodial area of the migrating macrophages (Figures 7, 8). In 3O-C_12_-HSL-stimulated macrophages, within their lamellipodial region, we found distinct longitudinal wave patterns of AQP9 intensity, being placed perpendicularly to the direction of suggested cell movement and containing the regions of AQP9 compression and rarefaction (Figures 8A–C).

In recent works, we and others have raised the possibility that AQP are important for host cell function during an infection. Thus, it was shown that AQP9 is upregulated in leukocytes after *in vivo* LPS stimulation in humans (Talwar et al., 2006). In alveolar epithelia, expression of AQP5 is critical for their mucin production and suggested to be a protective against *P. aeruginosa* infection (Zhang et al., 2011). Very recently, we have also found that *P. aeruginosa* does increase the AQP9 expression in macrophages, and that bacteria with a fully functional QS-system provoke a larger increase compared to a mutant strain lacking 3O-C_12_-HSL and C_4_-HSL (Holm et al., 2015).

In this study, we have disclosed that *P. aeruginosa* 3O-C_12_-HSL plays a critical role in the manipulation of an array of AQP9 characteristics, and thereby via water fluxes through AQP9 affects cell volume, area, shape, and protrusion development being important for proper macrophage functioning during infection. It is noteworthy, that the events in such an interplay between bacteria and macrophage might have an influence on the early onset of infection and inflammation and development of disease.

## Author contributions

AH planned and carried out experiments, analyzed the data, evaluated results and wrote the paper. KM evaluated results and prepared the paper. EV is PI of the project and designed the study, planned and did experiments, evaluated results, wrote and finalized the paper.

### Conflict of interest statement

The authors declare that the research was conducted in the absence of any commercial or financial relationships that could be construed as a potential conflict of interest.

## Supplementary material

Figure S1 **Quantification of cell area in 3O-C_12_-HSL-stimulated macrophages in individual experiments**. Cells were treated with 0.02% DMSO (Control), 10 or 50 μM 3O-C_12_-HSL, 25 μM HTS13286, or both 50 μM 3O-C_12_-HSL and 25 μM HTS13286, and a time-lapse Videos S1-S5 were recorded using the JULI microscope and cell area was quantified and shown as percent (%) change relative to the initial area. Values are the mean ± SE; the graphs show 8 individual experiments performed at separate days from 8 different donors (Donors I-VIII) and based on 20 cells for each condition (color coded) and experiment.

Figure S2 **Quantification of AQP9 intensity profiles across the macrophages**. Cells were treated with 3O-C_12_-HSL, stained for AQP9 and analyzed by LSCM, as in Figure 4. AQP9 immunofluorescence intensity profiles were measured over the cell in the direction of polarization as indicated by the white arrow in Figure 4. Quantification of AQP9 intensity in percentage of the cell length in macrophages after 3O-C_12_-HSL-treatments for 1, 4, and 24 h are shown in **(A)**, **(B)**, and **(C)**, respectively. Values are the mean ± SE. Data are from at least 4 different experiments performed on separate days from six different donors, and based at least 100 cells in total per condition were analyzed.

Video S1 **A time-lapse video of macrophages treated with 0.02% DMSO (Control)**.

Video S2 **A time-lapse video of macrophages treated with 10 μM 3O-C_12_-HSL**.

Video S3 **A time-lapse video of macrophages treated with 50 μM 3O-C_12_-HSL**.

Video S4 **A time-lapse video of macrophages treated with 25 μM HTS13286**.

Video S5 **A time-lapse video of macrophages treated with 50 μM 3O-C_12_-HSL and 25 μM HTS13286**.

Table S1 **Statistical analysis of cell area changes as shown in Figure 1**. Significant differences were analyzed by one-tailed unpaired Student's *t*-test and are indicated with: ^*^ when compared against control; § when compared between 10 μM 3O-C_12_-HSL and 50 μM 3O-C_12_-HSL; ¤when compared between 50 μM 3O-C_12_-HSL and 50 μM 3O-C_12_-HSL+HTS13286.

## Abbreviations

2D: two dimensional
3D: three dimensional
3O-C_12_-HSL: *N*-3-oxo-dodecanoyl-*L*-homoserine lactone
C_4_-HSL: *N*-butyryl-*L*-homoserine lactone
AHL: *N*-acylhomoserine lactone
AQP: aquaporin(s)
DMSO: dimethylsulfoxide
GAPDH: glyceraldehyde-3-phosphate dehydrogenase
KRG: Krebs-Ringer Glucose buffer
complete KRG: calcium-containing Krebs-Ringer Glucose buffer
LPS: lipopolysaccharide
LSCM: laser scanning confocal microscopy
NA: numerical aperture
PBS: phosphate buffered saline
RT: room temperature
STED: stimulated emission depletion nanoscopy
QS: quorum sensing
TNF: tumor necrosis factor.
