# Supplementary figures and images for: Pseudomonas aeruginosa N-3-oxo-dodecanoyl-homoserine Lactone Elicits Changes in Cell Volume, Morphology, and AQP9 Characteristics in Macrophages
Source: Front Cell Infect Microbiol. 2016 Mar 24;6:32. doi: 10.3389/fcimb.2016.00032 (PMC4805602; doi:10.3389/fcimb.2016.00032)

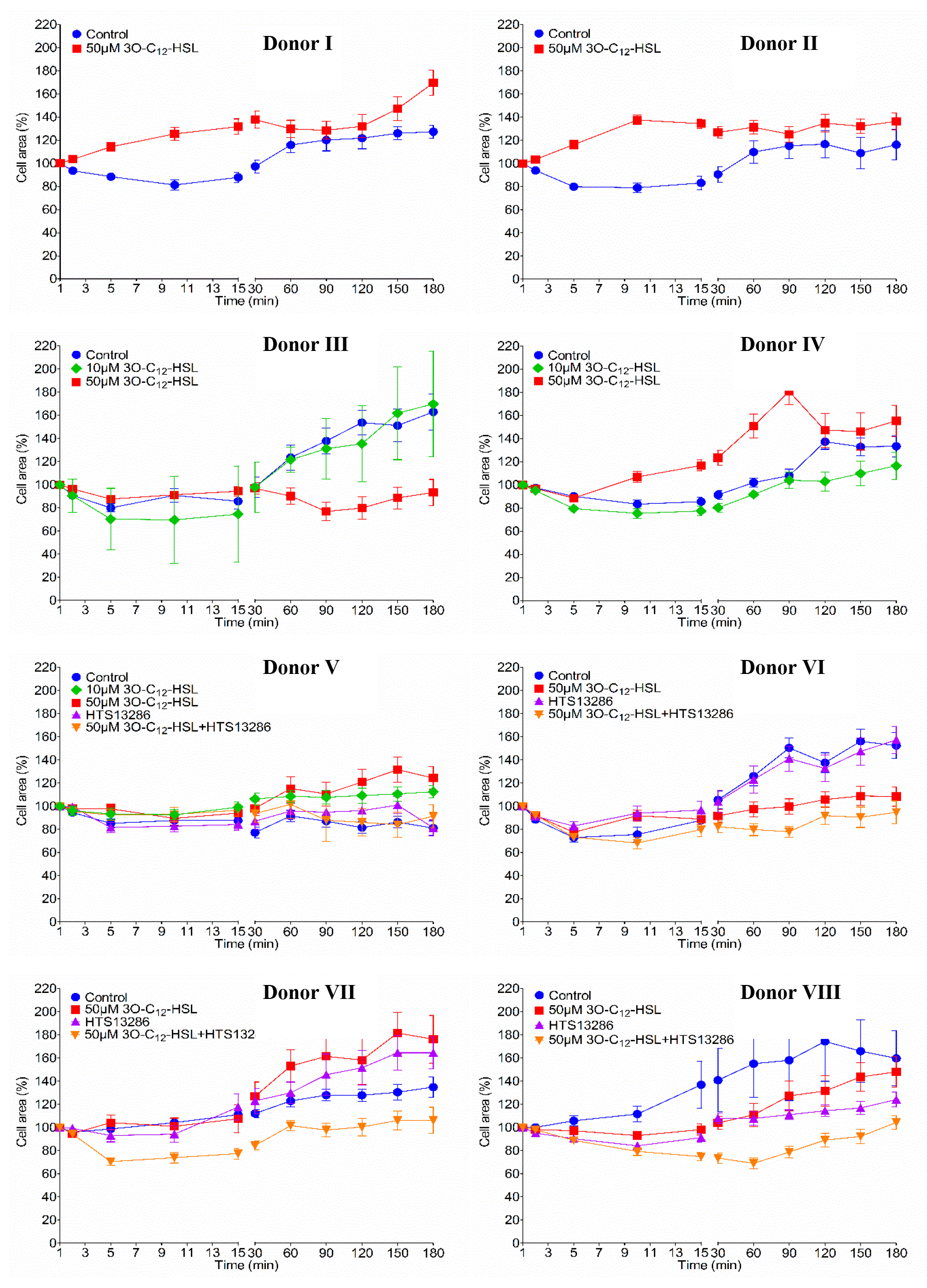

Supplement: Figure S1 — Quantification of cell area in 3O-C12-HSL-stimulated macrophages in individual experiments. Cells were treated with 0.02% DMSO (Control), 10 or 50 μM 3O-C12-HSL, 25 μM HTS13286, or both 50 μM 3O-C12-HSL and 25 μM HTS13286, and a time-lapse Videos S1-S5 were recorded using the JULI microscope and cell area was quantified and shown as percent (%) change relative to the initial area. Values are the mean ± SE; the graphs show 8 individual experiments performed at separate days from 8 different donors (Donors I-VIII) and based on 20 cells for each condition (color coded) and experiment. [file Image1.TIF]

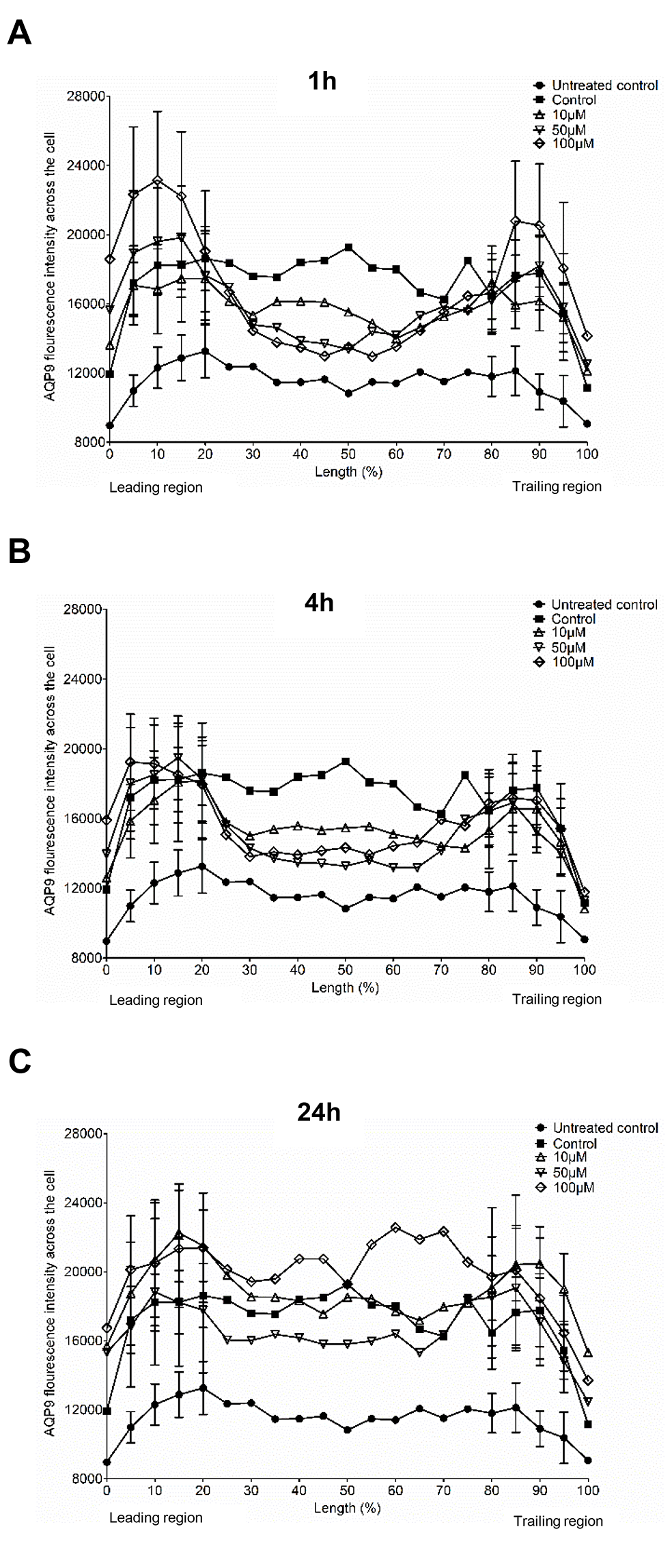

Supplement: Figure S2 — Quantification of AQP9 intensity profiles across the macrophages. Cells were treated with 3O-C12-HSL, stained for AQP9 and analyzed by LSCM, as in Figure 4. AQP9 immunofluorescence intensity profiles were measured over the cell in the direction of polarization as indicated by the white arrow in Figure 4. Quantification of AQP9 intensity in percentage of the cell length in macrophages after 3O-C12-HSL-treatments for 1, 4, and 24 h are shown in (A), (B), and (C), respectively. Values are the mean ± SE. Data are from at least 4 different experiments performed on separate days from six different donors, and based at least 100 cells in total per condition were analyzed. [file Image2.TIF]
