# Supplementary material for: Pseudomonas aeruginosa N-3-oxo-dodecanoyl-homoserine Lactone Elicits Changes in Cell Volume, Morphology, and AQP9 Characteristics in Macrophages
Source: Front Cell Infect Microbiol. 2016 Mar 24;6:32. doi: 10.3389/fcimb.2016.00032 (PMC4805602; doi:10.3389/fcimb.2016.00032)
Supplement: Table S1 — Statistical analysis of cell area changes as shown in Figure 1. Significant differences were analyzed by one-tailed unpaired Student's t-test and are indicated with: * when compared against control; § when compared between 10 μM 3O-C12-HSL and 50 μM 3O-C12-HSL; ¤when compared between 50 μM 3O-C12-HSL and 50 μM 3O-C12-HSL+HTS13286. [file DataSheet1.docx]

**Table S1. Statistical analysis of cell area changes as shown in Figure 1.**

| Time (min) | Cell area | | | | |
| --- | --- | --- | --- | --- | --- |
|  | Control | 10 µM 3O-C_12_-HSL | 50 µM 3O-C_12_-HSL | HTS13286 | HTS13286 +  50 µM 3O-C_12_-HSL |
| 1 | 2028 ±195 | 1846 ±91 | 2039 ±118 | 1655 ±207 | 1917 ±243 |
| 2 | 1922 ±184 | 1717 ±105 | 1995 ±138 | 1588 ±264 | 1863 ±248 |
| 5 | 1708 ±186 | 1518 ±215 | 1953 ±164 | 1387 ±228 | 1621 ±311 |
| 10 | 1698 ±174 | 1406 ±209 | 2134 ±195  **P*-value=0.05  § *P*-value=0.02 | 1412 ±171 | 1591 ±214 |
| 15 | 1823 ±190 | 1564 ±182 | 2220 ±181  § *P*-value=0.02 | 1599 ±177 | 1536 ±198  ¤ *P*-value=0.05 |
| 30 | 1983 ±204 | 1736 ±121 | 2341 ±208  §§ *P*-value=0.009 | 1816 ±238 | 1649 ±159  ¤ *P*-value=0.05 |
| 60 | 2335 ±221 | 1985 ±76 | 2533 ±204  § *P*-value=0.01 | 1969 ±285 | 1692 ±199  **P*-value=0.05  ¤ *P*-value=0.05 |
| 90 | 2464 ±170 | 2155 ±94  ** *P*-value=0.008 | 2572 ±242  § *P*-value= 0.04 | 2024 ±325 | 1692 ±208  **P*-value=0.04  ¤ *P*-value=0.03 |
| 120 | 2621 ±245 | 2134 ±108  * *P*-value=0.05 | 2731 ±25  § *P*-value=0.02 | 2090 ±325 | 1889 ±225  ¤ *P*-value=0.05 |
| 150 | 2608 ±244 | 2354 ±223 | 2861 ±213 | 2204 ±305 | 1986 ±247 |
| 180 | 2690 ±234 | 1. ±182   * *P*-value=0.01 | 2985 ±277 | 2220 ±364 | 2023 ±323 |

Significant differences were analyzed by one-tailed unpaired Student’s *t*-test and are indicated with: * when compared against control; § when compared between 10 µM 3O-C_12_-HSL and 50 µM 3O-C_12_-HSL; ¤ when compared between 50 µM 3O-C_12_-HSL and 50 µM 3O-C_12_-HSL+HTS13286.
